# Competition for Materno-Fetal Resource Partitioning in a Rabbit Model of Undernourished Pregnancy

**DOI:** 10.1371/journal.pone.0169194

**Published:** 2017-01-03

**Authors:** Jorge Lopez-Tello, Maria Arias-Alvarez, Maria Angeles Jimenez-Martinez, Rosa Maria Garcia-Garcia, Maria Rodriguez, Pedro Luis Lorenzo Gonzalez, Ruben Bermejo-Poza, Antonio Gonzalez-Bulnes, Pilar Garcia Rebollar

**Affiliations:** 1 Department of Animal Production, Veterinary Faculty, Complutense University of Madrid, Ciudad Universitaria, Madrid, Spain; 2 Department of Animal Medicine and Surgery, Veterinary Faculty, Complutense University of Madrid, Ciudad Universitaria, Madrid, Spain; 3 Department of Physiology (Animal Physiology), Veterinary Faculty, Complutense University of Madrid, Ciudad Universitaria, Madrid, Spain; 4 Department of Agrarian Production, E.T.S.I.A.A.B. Polytechnic University of Madrid, Ciudad Universitaria, Madrid, Spain; 5 Comparative Physiology Group, SGIT-INIA, Avda. Puerta de Hierro, Madrid, Spain; University of Southampton, UNITED KINGDOM

## Abstract

The major goal of animal production is to obtain abundant and healthy meat for consumers. Maternal food restriction (MFR) is often applied in farms to reduce production costs. However, the suitability of MFR in livestock animals is questionable, as this management may compromise maternal fitness due to a severe negative energetic balance and can induce Intrauterine Growth Restriction (IUGR) and prenatal programming in the offspring. Here, we sought to determine, using pregnant rabbits, the consequences of MFR on maternal endocrine and metabolic status and conceptus development. Pregnant dams were distributed into three groups: CONTROL (*ad libitum* feeding throughout the entire pregnancy; mean pregnancy length being around 31 days), UNDERFED (50% MFR during the entire pregnancy) and EARLY-UNDERFED (50% MFR only during the preimplantation period, Days 0–7). Maternal leptin concentrations and glycemic and lipid profiles were determined throughout pregnancy, whilst conceptus development was assessed *ex-vivo* at Day 28. Placental parameters were determined by macroscopic and histological evaluations and apoptotic assessments (TUNEL and Caspase-3). The main results of the study showed that, despite MFR altered maternal plasma lipid concentration (P<0.05), there were no effects on maternal bodyweight, plasma leptin concentration or glycemic profile. Fetal crown-rump lengths were reduced in both undernourished groups (P<0.001), but a significant reduction in fetal weight was only observed in the UNDERFED group (P<0.001). Growth in both undernourished groups was asymmetrical, with reduced liver weight (P<0.001) and significantly increased brain: fetal weight-ratio (P<0.001) and brain: liver weight-ratio (P<0.001) when compared to the CONTROL group. A significant reduction in placental weight was only observed in the UNDERFED group (P<0.001), despite both undernourished groups showing higher apoptotic rates at decidua and labyrinth zone (P<0.05) than the CONTROL group. Thus, these groups evidenced signs of placental degeneration, necrosis and stromal collapse. In summary, MFR may encourage the mother to make strategic decisions to safeguard her metabolic status and fitness at the expense of growth reduction in the litter, resulting in enhanced apoptotic and pathological processes at placental level and IUGR.

## Introduction

The major goal of animal production is to obtain abundant and healthy meat for consumers, which relies on adequate management of breeding animals and pregnancy periods [1]. In livestock animals, maternal nutrition has been largely recognized as a key factor for pregnancy success [1, 2]. Periods of maternal food restriction (MFR) during gestation can result in offspring suffering from Intrauterine Growth Restriction (IUGR), defined as the failure of a fetus to reach its genetic potential size [3]. The same situation has been found in humans, with MFR having a strong impact of IUGR occurrence and a higher incidence of non-communicable diseases, like obesity, cognitive dysfunctions or cardiometabolic disorders [4–6].

In livestock animals, the intensive productive rhythms and the different farm managements aiming to reduce productive costs have led to a higher occurrence of IUGR in these animals [1, 7]. Thereby, affecting the quality of their meat (muscle fibers and marbling), athletic performance or fleece production [2, 8] and lastly resulting in poorer incomes for the livestock producer and lower quality products for the consumers. Despite these inconveniences, MFR protocols applied in specific periods of the pregnancy, such as the preimplantation period, in which the embryo’s requirements are low and the mother presents an anabolic status, could reduce productive costs and be an alternative strategy in farms [9, 10]. However, the advantages and disadvantages of such managements need to be further investigated, since inadequate nutrition from early gestation can influence placentation processes (specifically the allocation of trophectoderm and inner cell mass within the blastocyst [11]), which may impair placental development and function [12], compromising pregnancy outputs. In fact, experimental studies suggest that impaired placental structure or function (*e*.*g*. placental insufficiency) may contribute to IUGR in response to undernutrition [12, 13]. Most of these studies have been performed in rodents [14], whilst the use of large animals is scarce. However, large animals (sheep, pig or rabbit) offer a wider range of benefits for the purpose of this assessment, as the results obtained from these trials, especially those based on MFR protocols, can be useful not only for biomedicine but also to unravel the aforementioned advantages and disadvantages of the application of MFR regimens to livestock animals.

In the last years, the rabbit, considered as a livestock animal in the Mediterranean area (meat and fibre production [15, 16]), has emerged as a valuable model to investigate IUGR [17–31], as compared to a sheep or a pig, this animal does not need large animal facilities and gestational length is shorter (term around Day 31; D31). Thus, the rabbit develops a discoid hemochorial placentation and the fetuses have an accelerated perinatal brain growth, such characteristics are comparable with the human [32, 33]. Moreover, hemodynamic changes occurring during pregnancy are also similar to the human, with an important increase in maternal blood pressure throughout gestation [32, 34]. In rabbits, the effects of MFR during gestation [17, 30, 31, 35–41] vary depending on the period of the pregnancy exposed, the level of the restriction applied and the capacity of the mother to compensate, particularly during late pregnancy when fetal growth is maximal [42]. When it comes to the mother, MFR is usually linked to metabolic and hormonal changes [35–37] and low milk production [38]. Meanwhile, in the fetus, hemodynamic alterations and poor biometric outcomes can be observed [17, 30, 31, 39–41].

It has been previously shown using other animal species that MFR based on preconception or in *ad libitum* food intake can affect placental development [43], induce IUGR [44] and developmental programming of adult diseases in the offspring [45]. In previous work, using the rabbit as a model, we evaluated the effects of MFR based on *ad libitum* pre-pregnancy intake [30, 31]. However, in these studies, MFR was applied when the rabbit embryo was already implanted (from D9 to term). MFR resulted in fetal IUGR (measured on day 28 of pregnancy and at birth) despite no differences in placental weight or in perinatal mortality were observed. Here, we sought to determine the consequences of MFR applied only during the critical period of preimplantation (D0-D7) or throughout the gestation (D0 onwards) on: 1) maternal food intake, endocrine and metabolic status of the dams, 2) conceptus development at term and 3) placental homeostasis determined by histopathological study and apoptosis quantification.

## Materials and Methods

### Animals and experimental design

The study was performed according to the Spanish Policy for Animal Protection RD53/2013. The experiment was specifically assessed and approved by the Polytechnic University of Madrid (UPM) Committee of Ethics in Animal Research, which is the named Institutional Animal Care and Use Committee (IACUC) for the UPM, and by the Community of Madrid, which is the authority in charge for animal research (Ref. PROEX 302/15). The does were housed at the animal facilities of the UPM, which meet the local, national and European requirements for Scientific Procedure Establishments. Prior to the experimental phase (two weeks before mating), a total of 32 New Zealand x California rabbits (average 4.74 ± 0.12 Kg) were fed *ad libitum* with a diet containing 16% crude protein, 37% crude fiber, 3.7% fat and 2400 kcal/kg of crude energy (NANTA, Madrid, Spain). During this period the food consumption of each dam was recorded daily. Dams were inseminated (D0 of pregnancy) with fresh diluted semen (commercial extender, MA 24, Ovejero, León, Spain). Each dose contained at least 25 million spermatozoa in 0.5 ml of diluent (Magapor S.L., Zaragoza, Spain). Ovulation was induced with gonadoreline at the time of mating (20 μg/doe, i.m.; Inducel-GnRH, Ovejero, León, Spain). At this moment dams were randomly allocated into three groups: *Ad libitum* feeding along the pregnancy (CONTROL; n = 9), 50% restriction of their previous *ad libitum* intake throughout pregnancy (UNDERFED; n = 12) or restricted only during the preimplantation period (D0-D7; ≈22% of the total pregnancy) followed by *ad libitum* feeding until the end of pregnancy (EARLY-UNDERFED; n = 11). Food intake was also recorded weekly in all experimental groups during gestation.

### Maternal blood sample collection

Evaluation of metabolic parameters and hormones was performed in five dams per experimental group. Blood was obtained weekly (D0, D7, D14, D21, D28) by ear-puncture. Blood was placed in tubes with Ethylenediaminetetraacetic acid (EDTA) as anticoagulant and centrifuged for 15 min at 1200 g to obtain ≈2 ml of plasma per dam. Concentrations of leptin were determined in a single analysis using the Multi-species Leptin RIA kit (Demeditec Diagnostics GmbH, Kiel, Germany). The assay sensitivity was 1.0 ng/ml and the intra-assay variation coefficient was 3.1%. Parameters relating to the glycemic (glucose and fructosamine) and lipid metabolisms (total cholesterol, high-density lipoproteins cholesterol [HDL-c], low-density lipoproteins cholesterol [LDL-c] and triglycerides) were measured on a clinical chemistry analyzer according to the manufacturer’s instructions (Saturno 300 plus. Crony Instruments s.r.i., Rome, Italy).

### Macroscopic study of the fetuses and placentas

**Fig 1:**
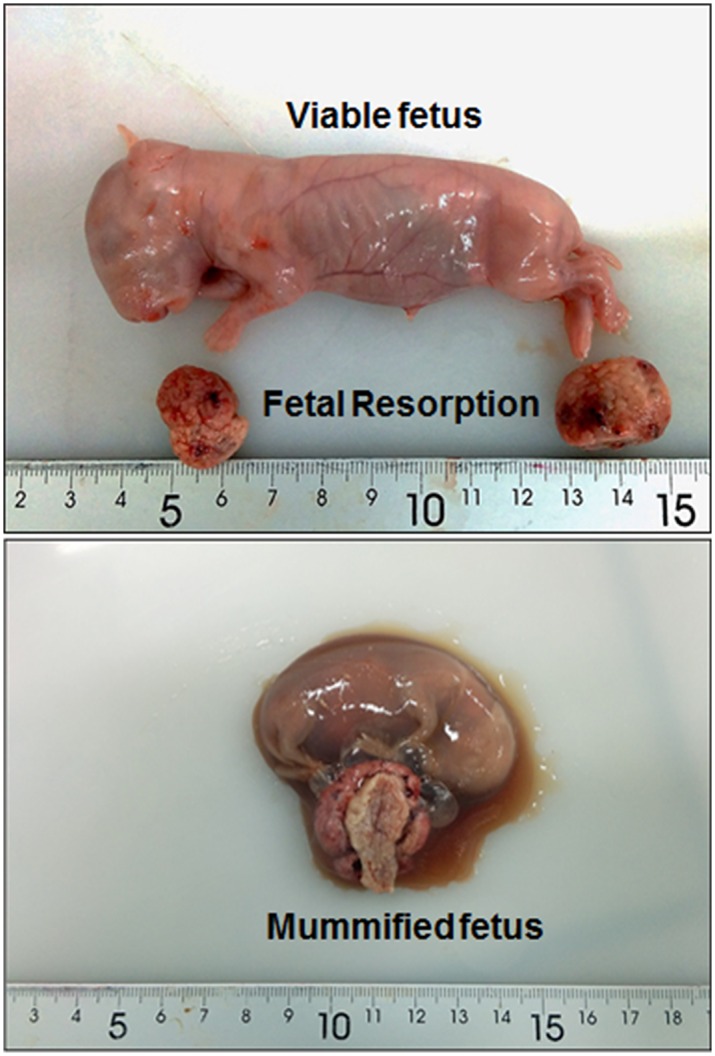
Macroscopic images of rabbit conceptus at D28 of pregnancy.

Dams were weighed and euthanized (30 mg/kg, IV marginal ear vein administration; Dolethal, Madrid, Spain) at D28. The gravid uterus was removed by a medial laparotomy and subsequently weighed. Fetuses were dissected from their extraembryonic membranes and classified according to the following criteria: viable (with a correct morphology and weight for the gestational age), mummified (dead in uterus with signs of shrivel and drying) or fetal resorption (residual placental tissue attached to the endometrium) (Fig 1). The implantation rate was estimated as the ratio between total numbers of implanted fetuses and *corpora lutea* present in the ovaries. In viable fetuses, crown-rump length and bodyweight were determined by the use of scales and calipers prior to decapitation, after which brain and liver tissue were weighed. Brain and liver to fetal weight-ratios and brain to liver weight-ratio were obtained as indexes for IUGR [18, 46]. Finally, placentas from viable fetuses (except those selected for histopathological study) were weighed and decidua and labyrinth sections were separated and characterized by weight, length, breadth and thickness with scales and calipers. Placental efficiency was calculated as fetal to placental weight-ratio [47, 48].

### Microscopic study of placenta

The placentas closest to the left ovary were cut in half, fixed in 4% paraformaldehyde and switched to 70% ethanol the following day. Samples [CONTROL (n = 8; one placenta was excluded for technical reasons), EARLY-UNDERFED (n = 11), UNDERFED (n = 12)] were embedded in paraffin and sectioned at 4 μm thickness with a semi-automated rotary microtome (Leica, Wetzlar, Germany).

The hstopathological study of placenta was performed by staining all the samples with hematoxylin and eosin and analyzing them under a light microscope (Olympus BX40, Hamburg, Germany) by a trained pathologist blinded to the experimental groups. Stromal collapse of the labyrinth and decidual sclerosis of vascular channels were each graded and scored using the following criteria: unremarkable = 0, mild = 1, moderate = 2 and severe = 3. Other descriptive pathological features such as necrosis, mineralization and inflammatory infiltrates were recorded. The width of each placental layer (decidua, labyrinth and junctional zone) was analyzed by measuring five random fields of each layer under a 1.25x objective and obtaining the mean value.

The degree of apoptosis was determined using the ApopTag *in situ* apoptosis detection kit (Millipore Corp., San Francisco, USA) for terminal deoxynucleotidyl transferase dUTP-mediated nick-end labeling (TUNEL), according to the manufacturer's instructions and as previously described [49]. Sections were deparaffinized and treated with proteinase K (Roche Diagnostics GmbH, Mannheim, Germany). Endogenous peroxidase activity was blocked with 3% hydrogen peroxide in phosphate-buffered saline (PBS), and incubated with anti-digoxigenin conjugate in a humidified chamber at room temperature for 30 min. Sections were counterstained with methyl green. Ten random fields of each placental zone were photographed under a 20x objective and quantified using Image J software. The percentage of apoptotic cells was estimated as the number of TUNEL stained nuclei divided to the total number of total stained nuclei per zone × 100 [49].

To corroborate the results obtained from TUNEL assessment, we also performed Caspase-3 quantification. In brief, endogenous peroxidase activity was blocked by a 30 min treatment with 3% hydrogen peroxidase in absolute methanol. Nonspecific binding was blocked by incubating the sections in 5% normal goat serum (sc-2043, Santa Cruz Biotechnology, Santa Cruz, CA, USA) and incubated overnight with the primary antibody (1:25; caspase-3 antibody ab2171; Abcam, Cambridge, UK). After subsequent washes, sections were incubated with biotinylated anti-mouse secondary antibody (1:200; Santa Cruz Biotechnology, Santa Cruz, CA, USA) and subsequently with the avidin-biotin complex (ABC Vector Elite kit, Vector Laboratories, Burlingame, CA, USA). After chromogen incubation (Vector Nova RED substrate Kit for Peroxidase, Vector Laboratories, Burlingame, CA, USA), sections were counterstained with hematoxylin and analyzed under a light microscope (Leica DM IL, Wetzlar, Germany). The percentage of Caspase-3 positive immunostaining was determined as caspase-3 positive stain in a zone (pixels)/total surface of that zone (pixels) x 100.

### Statistical analysis

Statistical analysis was performed with SAS software (Statistical Analysis System Institute Inc., Cary, NC, USA). The effects of MFR in food intake, leptin and plasma metabolic parameters were evaluated by two-way analysis of variance (ANOVA). Maternal bodyweight and parameters related to conceptus development were analyzed by one-way ANOVA with maternal bodyweight at the beginning of the trial and number of fetuses per dam as covariates, respectively. If significant main effects were detected, T test (for parametric variables) or Kruskal-Wallis test (non-parametric variables) were used to compare averages among groups. Histological findings related to percentages were obtained by a Chi square test. All data were reported as mean±SEM and probabilities were considered significant at P<0.05.

## Results

### Early-MFR induced maternal food intake compensation in later gestation and circumscribed changes in lipid metabolism without affecting bodyweight, leptin concentrations or glycemic profile

**Fig 2:**
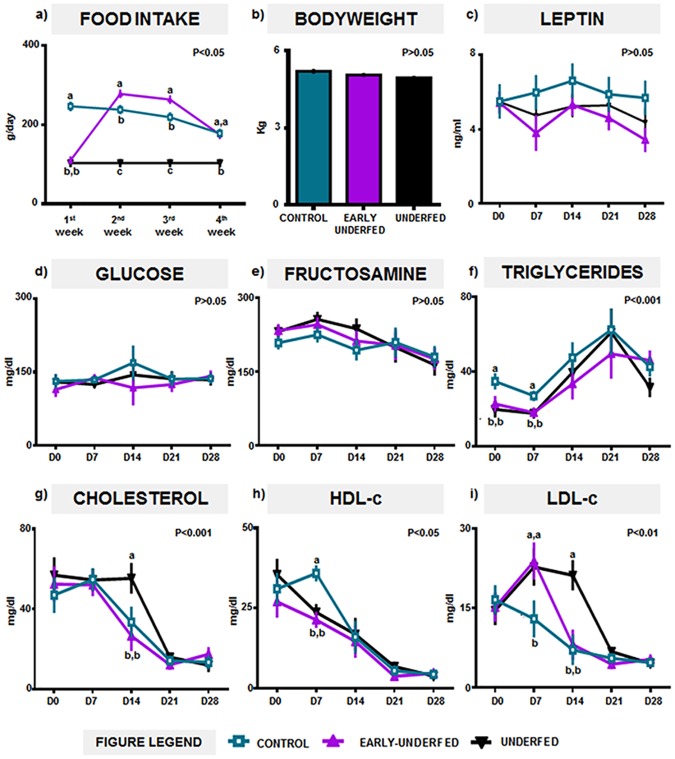
Effects of MFR on maternal food intake, bodyweight and plasma leptin, glucidic and lipid concentrations during pregnancy. Number of pregnant dams per group employed for Fig. a) and b): CONTROL = 9, EARLY-UNDERFED = 11, UNDERFED = 12; Number of pregnant dams per group employed for Fig. c) to i): CONTROL = 5, EARLY-UNDERFED = 5, UNDERFED = 5. Statistical analyses were performed by ANOVA. Data presented as mean±SEM. Different superscripts indicate significant differences between groups (P<0.05).

Both undernourished groups had similar food intake during the 1^st^ week of pregnancy and showed significant differences compared with the CONTROL group (Fig 2a). Once the restriction finished, EARLY-UNDERFED group significantly increased its food intake compared to the CONTROL group during the 2^nd^ and 3^rd^ weeks of pregnancy, but not during the last week of gestation, in which both groups significantly decreased their food intake (Fig 2a). These changes in dietary patterns were not associated to changes in the bodyweight of the dams at D28 calculated after retrieval of the pregnant uterus (Fig 2b). Furthermore, MFR did not affect either plasma leptin concentrations (Fig 2c), glucose or fructosamine levels (Fig 2d and 2e), but did alter the circulating lipid metabolites in early to mid-gestation (D0 to D14). In this regard, during the preimplantation period (D0 to D7) higher concentrations of triglycerides were found in the CONTROL group compared to both undernourished groups (Fig 2f). Meanwhile, plasma cholesterol concentrations were only significantly higher in the UNDERFED group on D14 *versus* the CONTROL and EARLY-UNDERFED groups (Fig 2g). On D7, plasma HDL-c concentrations were significantly reduced in both undernourished groups (Fig 2h) despite the fact that within the same period of gestation, LDL-c concentrations increased in these two groups respect CONTROL values (Fig 2i). Lastly, LDL-c concentrations remained higher only in the UNDERFED group until D14 compared to EARLY-UNDERFED and CONTROL groups (Fig 2i).

### MFR disrupted fetal growth trajectory but did not alter the number of viable fetuses at D28

MFR reduced the weight of the gravid uterus in the UNDERFED group compared to the CONTROL and EARLY-UNDERFED groups, despite no differences in the number of fetuses per litter or in implantation rate (Table 1). Fetuses in the UNDERFED group showed the lowest crown-rump length and fetal weight (total, head and trunk) while the EARLY-UNDERFED group only showed a significant reduction of crown-rump length when compared to the CONTROL group. Brain and liver weight were significantly lower in the UNDERFED group when compared to the CONTROL and the EARLY-UNDERFED groups (Table 1). In the EARLY-UNDERFED group, liver weight was significantly lower than in the CONTROL group. However, no difference was found in the absolute weight of the brain between these two groups. The assessment of brain ratio showed a significant increase in both undernourished groups, while liver ratios were equally reduced in EARLY-UNDERFED and UNDERFED compared to the CONTROL. Thus, brain: liver weight ratio was higher in both restricted groups with respect to the CONTROL group.

**Table 1:** Effects of MFR on conceptus development from CONTROL, EARLY-UNDERFED or UNDERFED groups. Data obtained from 9 to 12 dams; Number of fetuses per group: CONTROL n = 105, EARLY-UNDERFED n = 126, UNDERFED n = 117. Data presented as mean±SEM. Different superscripts indicate significant differences between groups (P<0.01). NS non-significant differences.

	CONTROL	EARLY-UNDERFED	UNDERFED	P
**Litter size**				
Gravid uterus (g)	762.96±52.82^a^	757.65±47.78^a^	572.19±45.74^b^	<0.001
Total number of fetuses (n)	12.11±1.20	11.90±1.09	10.33±1.04	NS
Viable fetuses (n)	11.66±1.16	11.18±1.08	9.75±1.00	NS
Mummified fetuses (n)	0.22±0.19	0.36±0.17	0.16±0.16	NS
Resorptions (n)	0.22±0.20	0.36±0.18	0.41±0.17	NS
Implantation rate (%)	88.69±9.06	92.27±8.01	93.37±7.63	NS
**Fetuses**				
Crown-Rump length (cm)	9.90±0.09^a^	9.45±0.08^b^	9.13±0.08^c^	<0.001
Fetal weight (g)	42.10±0.67^a^	41.10±0.60^a^	35.67±0.63^b^	<0.001
Head weight (g)	10.08±0.12^a^	9.78±0.10^a^	8.87±0.11^b^	<0.001
Trunk weight (g)	31.48±0.54^a^	30.89±0.48^a^	26.25±0.51^b^	<0.001
Brain weight (g)	1.03±0.01^a^	1.06±0.01^a^	0.96±0.01^b^	<0.001
Brain ratio (%)	2.49±0.04^a^	2.63±0.04^b^	2.77±0.04^c^	<0.001
Liver weight (g)	3.59±0.09^a^	3.21±0.08^b^	2.68±0.09^c^	<0.001
Liver ratio (%)	8.42±0.15^a^	7.60±0.14^b^	7.41±0.14^b^	<0.001
Brain:Liver ratio	0.31±0.01^a^	0.37±0.01^b^	0.39±0.01^b^	<0.001
**Placenta**				
Placenta Efficiency	7.0±0.12^a^	6.76±0.11^a^	7.51±0.12^b^	<0.001
Total placenta weight (g)	5.99±0.15^a^	5.91±0.14^a^	4.67±0.15^b^	<0.001
**Decidua**				
Weight (g)	1.56±0.05^a^	1.52±0.04^a^	1.22±0.04^b^	<0.001
Length (cm)	3.55±0.07^a^	3.72±0.07^a^	3.24±0.07^b^	<0.001
Breadth (cm)	1.43±0.04	1.38±0.03	1.37±0.04	NS
Thickness (cm)	0.40±0.02^a^	0.37±0.01^a^	0.31±0.01^b^	<0.001
**Labyrinth zone**				
Weight (g)	4.12±0.12^a^	3.88±0.11^a^	3.13±0.12^b^	<0.001
Length (cm)	3.66±0.05^a^	3.57±0.05^a^	3.28±0.05^b^	<0.001
Breadth (cm)	2.68±0.06^a^	2.60±0.06^a^	2.35±0.06^b^	<0.001
Thickness (cm)	0.52±0.02^a^	0.49±0.02^ab^	0.46±0.02^b^	<0.01

### MFR altered placental development and resulted in histopathological changes and higher apoptotic levels

Placental development was altered in both undernourished groups with a more severe effect when MFR was carried out throughout gestation. MFR reduced placental weight only in the UNDERFED group although a compensatory placental efficiency was observed (measured as fetus-to-placenta weight-ratio; Table 1). Decidua and labyrinth sizes were reduced in this group except for the decidua breadth, which did not show significant differences among groups. In the EARLY-UNDERFED group, labyrinth thickness showed intermediate values between the CONTROL and UNDERFED groups. No other differences were observed between groups within the remaining macroscopic parameters measured.

**Fig 3:**
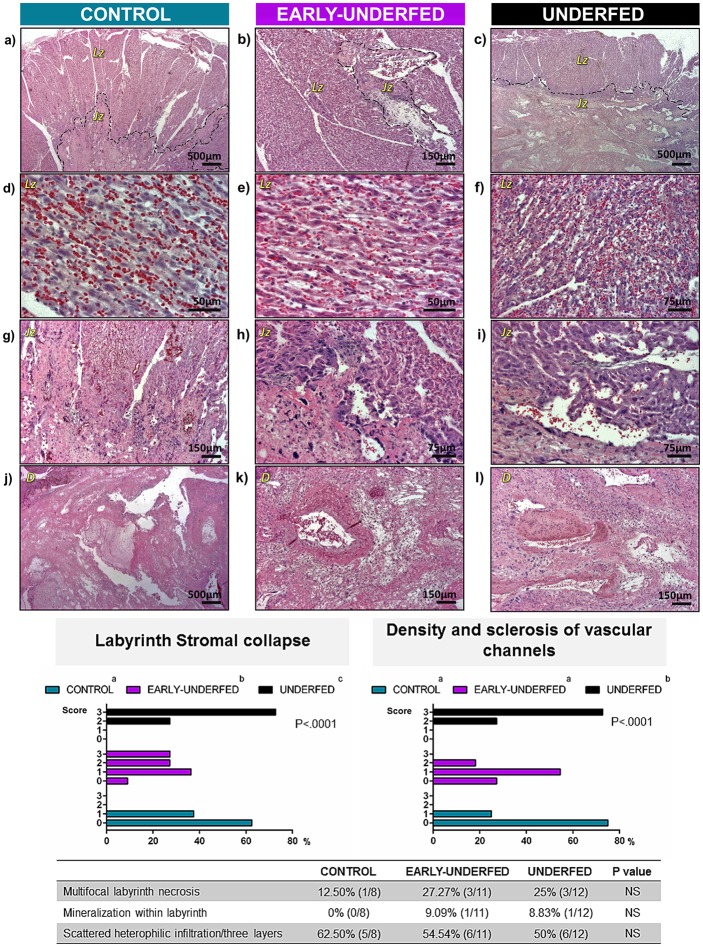
Histological findings of the rabbit placenta at D28 of pregnancy in CONTROL, EARLY-UNDERFED and UNDERFED groups. Figures A to C: Labyrinth (Lz) and junctional (Jz) zones of the rabbit placenta in the three experimental groups. (D & E) CONTROL AND EARLY-UNDERFED groups. Densely cellular labyrinth with endothelium lined vascular channels and trophoblasts. (F) Labyrinth of the UNDERFED group with variably spaced endothelium lined vascular channels separated by collagen fibers (stromal collapse) and trophoblasts. Figures G to I: Junctional zone in the three experimental groups. (G) CONTROL group. Dense connective tissue matrix supporting stromal cells, trophoblasts and blood capillaries that extend into the labyrinth. (H) EARLY-UNDERFED group. Dense connective tissue containing stromal cells and trophoblasts. (I) UNDERFED group. Dense connective tissue slightly thinner and supporting fewer stromal cells and trophoblasts than the two other experimental groups. The overlaying labyrinth contains moderately spaced vascular channels with decreased cellularity. Figures J to L: Decidua (D) in the three experimental groups. (J) CONTROL GROUP. Normal decidua with large vascular sinuses, stroma, fibrin and necrosis. (K) EARLY-UNDERFED group. Large vessel within the decidua surrounded by abundant edematous stroma. (L) UNDERFED group. Markedly thinned decidua layer with spaced vessels containing hyalinised walls, a thrombus, and surrounded by dense stromal collagen. Number of placentas per group: CONTROL = 8, EARLY-UNDERFED = 11, UNDERFED = 12; Statistical analyses were performed by Chi square test. In the charts and table different superscripts indicate significant differences between groups (P<0.0001), NS non-significant differences.

Significant histological changes are summarized in Fig 3. The most notable changes in the placenta occurred in the UNDERFED group, which showed the lowest decidual width value (2.04 ± 0.32 mm) compared to the CONTROL (3.19 ± 0.32 mm) and the EARLY-UNDERFED groups (3.08 ± 0.29 mm) (P<0.05). Thus, the decidua of the UNDERFED group had fewer vessels, most of which contained hyalinized and sclerotic walls and lesser supporting stroma than the other groups (Fig 3). The labyrinth stroma in both restricted groups was significantly reduced in width compared to the CONTROL group (2.46 ± 0.14, 2.04 ± 0.19 and 3.03 ± 0.20 mm, for EARLY-UNDERFED, UNDERFED and CONTROL groups, respectively; P<0.05). The reduced width corresponded with the stromal collapse of the labyrinth which contained lesser cellularity (vascular endothelium and trophoblasts) than the CONTROL group. Thus the UNDERFED group showed moderate to severe signs of collapse followed by the slightly less severe EARLY-UNDERFED group (Fig 3). Necrosis was observed in all experimental groups without significant differences (Fig 3). Mild mineralization was associated with the necrosis and was likewise insignificant between groups (Fig 3). The width of the junctional zone was similar between groups (CONTROL: 0.42 ± 0.08 mm; EARLY-UNDERFED: 0.35 ± 0.07 mm and UNDERFED: 0.30 ± 0.09 mm; P>0.05). Mild heterophilic inflammation was observed in the three layers of all the experimental groups and was within normal limits for this organ (Fig 3).

**Fig 4:**
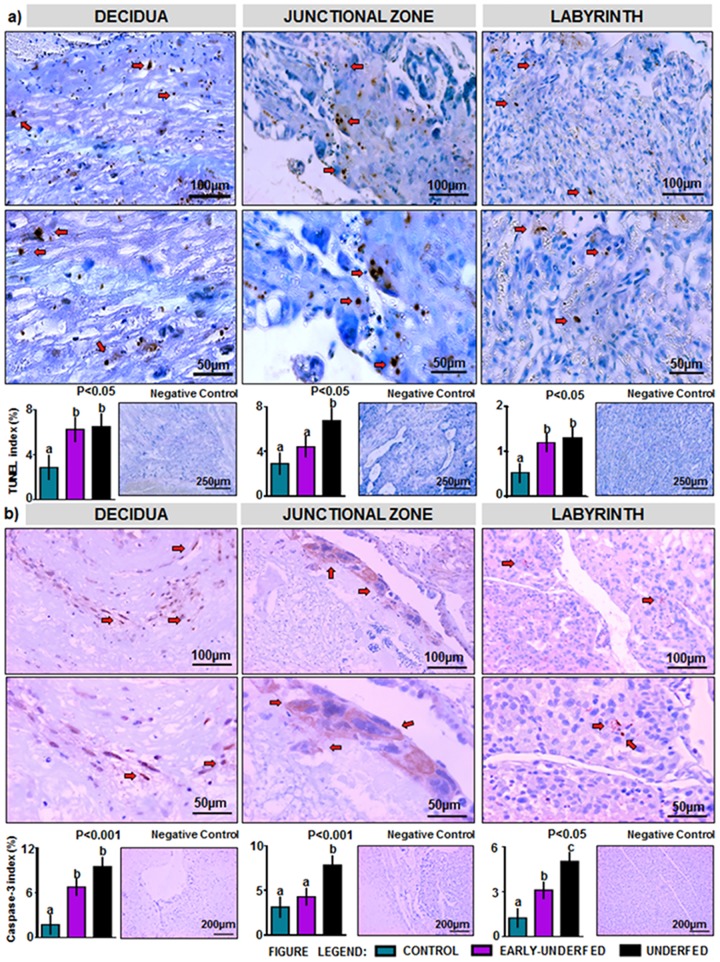
Apoptosis assessments of the rabbit placenta at D28 of pregnancy. (a) Apoptosis quantified by TUNEL (positive staining) in decidua, junctional zone and labyrinth. (b) Localization and percentage of Caspase-3 (positive immunostaining) in decidua, junctional zone and labyrinth. Number of placentas per group: CONTROL = 8, EARLY-UNDERFED = 11, UNDERFED = 12; Statistical analyses were performed by ANOVA. Data presented as mean±SEM. Different superscripts in the charts indicate significant differences between groups (P<0.05).

Concerning placental apoptotic rate, higher rates determined by TUNEL assay were found in both undernourished groups at the decidua and labyrinth zones (Fig 4a). At the junctional zone, only the UNDERFED group evidenced a significant higher rate when compared to the EARLY-UNDERFED and CONTROL groups (Fig 4a). Results from the assessment of caspase-3 levels (Fig 4b) were similar to those found in TUNEL assay in decidua and junctional zone. In contrast, the labyrinth showed higher rates of immunostaining in the UNDERFED group followed by the EARLY-UNDERFED group and compared to the CONTROL group.

## Discussion

**Fig 5:**
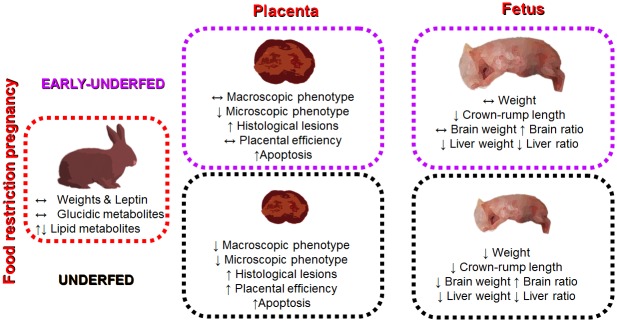
Summary illustration showing the effects of MFR in the EARLY-UNDERFED and UNDERFED groups on placental and fetal outcome.

The current study shows that MFR applied to pregnant rabbits, despite the absence of significant changes in maternal bodyweight, plasma leptin concentration or glucidic metabolites, affects maternal lipid concentrations during early to mid-gestation but not during late gestation, when fetal growth is exponential. MFR applied only during the preimplantational period did not result in low fetal weight, but was enough to disrupt crown-rump length and impair organogenesis. Consistent with these findings, severe alterations on placental and fetal development can be observed when MFR is maintained throughout gestation (Fig 5).

The lack of effects of MFR on maternal bodyweight found in the current study supports previous studies in rabbits [30, 38, 41] and differs from other animal species in which the application of MFR resulted in maternal bodyweight loss, like rodents [50–53], sheep [54, 55] and primates [56, 57]. These differences between species and experimental studies may be related to feeding management (*ad libitum* feeding *vs* maintenance requirements), fat stores prior to the experimental phase, or may be as well associated to reproductive strategies adopted by the mothers [58]. In this sense, undernourished dams had similar implantation rates and litter sizes compared to the CONTROL group. These results may suggest that undernourished mothers do not invest more than indispensable in their offspring growth, as a preventive strategy for future gestations and for their own energetic balance [58]. Despite the fact that offspring growth was affected, undernourished females could have accumulated resources to deal with the lactation, to guarantee a better offspring feeding, postnatal survival and growth and therefore ensure the transmission of maternal genes to future generations [58].

Concomitantly, plasma leptin concentrations did not decline in any of the groups in our study, opposite to previous works in rabbit [37], mouse [51, 59] and sheep [55]. We hypothesize that the absence of differences in our study may be explained by different facts. At first glance, the most obvious reason could be related to the number of animals sampled. However, sample size was calculated according to the study of Menchetti et al. [37], which found variations in leptin profile using a similar number of pregnant rabbits. Other explanations may involve an increased production of leptin either by maternal, placental or fetal tissues [52, 60]. This hypothesis may reinforce the idea that despite the restriction, food amount could be enough to satisfy maternal and fetal requirements. Expression of leptin-RNA in maternal adipose and fetoplacental tissues should corroborate this idea. Lastly, leptin concentrations could be influenced by hormonal milieu, specially insulin and/or glucose [61], estrogens [62] or glucocorticoids, which have been shown to induce leptin expression [63].

In contrast, maternal plasma lipids changed but only in early to mid-gestation. Such period of pregnancy can be considered as an anabolic state, due to the low requirements of the developing offspring [64, 65]. In contrast, we did not observe any variations in lipid profile during the last third of gestation, a period of accelerated breakdown of fat depots to satisfy fetal demands [66, 67]. In this regard, plasma cholesterol and LDL-c concentrations increased in the undernourished groups during the period comprising D7 to D14 of pregnancy, suggesting a certain level of fat mobilization may be needed to deal with the implantation process [68], yolk sac formation and the establishment of the chorio-allantoic placental circulation [69]. In contrast, HDL-c concentrations raised in the CONTROL group to return excess of cholesterol from peripheral tissues back to the liver in response to an adequate level of resources [70]. Furthermore, maternal triglycerides concentrations were lower in both undernourished groups which may be a mechanism to increase deposition of fat in maternal adipose tissue for lactation [71]. Overall, these findings may reinforce, once more, the aforementioned strategies adopted by the mothers to constrain allocation of resources during late gestation on behalf of themselves and future lactation.

Following this argument, the placenta is central to this “tug of war” over nutrient allocation as it is the surface area for exchange between mother and fetus [58; 72]. Experiments based on genetic and dietary manipulations have demonstrated that the placenta interprets fetal and maternal interests, adapting its phenotype and function according to resource availability [72–74]. An example of these fascinating adaptations is that undernourished placentas can overcome MFR and maintain or even increase their nutrient transfer capacity to help maintain fetal growth [50]. In our study, placental weight was reduced in the UNDERFED group. Such finding supports the hypothesis that the preimplantational period may be a critical timing for placental establishment, since the same level of MFR applied after implantation (D9 of gestation in rabbits) did not reduce placental weight [31].

Conversely, no gross macroscopic differences were found in the EARLY-UNDERFED, reinforcing previous findings in sheep that were only restricted during early-mid gestation, and then returned to normal feeding [75]. Interestingly, the microscopic assessment of placental thickness showed a significant reduction in labyrinth expansion in both undernourished groups, which supports previous work [35]. The progressive reduction in the surface area of the labyrinth may affect placental nutrient transporters and contribute to the poor fetal outcome. In rats, MFR by 50% downregulated *GLUT3* transporter expression, resulting in offspring with IUGR [76]. Furthermore, in a primate model of MFR [77], expression of glucose and amino acid transporters (*GLUT1*, *TAUT*, *SNAT2*, *LAT1*, and *LAT2*) were reduced as well as placental and fetal weights. Future work should evaluate such findings in our rabbit model of MFR and its connection with IUGR.

The reduction in the size of the decidua only observed in the UNDERFED group could be associated to two different processes. Firstly, a reduced trophoblast invasion could have impaired the correct remodeling process of the spiral arteries. Thus, the high incidence of sclerotic processes in the decidual vascular bed only observed in the UNDERFED group could be linked to the high circulating levels of cholesterol observed in this group on D14. In this sense, lipid peroxides and oxygen radicals can alter endothelial cells, resulting in fibrin deposition in the vessel walls [67]. Consequently, the arterial sinuses system in the rabbit, which can be identified within this period of gestation [78] and is responsible to retain maternal blood flow and then supply it to fetal area could be altered, resulting in IUGR. On the other hand, the reduction in decidua size could be a mechanism of these placentas to support fetal demands for growth by depletion of their glycogen reserves allocated in the small uninucleated cells of this placental section [79], and therefore may explain the increase in placental efficiency and the reduction in weight. However, this last effect remains speculative in this study; further investigations should corroborate this hypothesis and determine placental glycogen storage in restricted placentas of rabbit dams, as glycogen reserves in the murine placenta are an important source of glucose in the final stages of gestation [80]. However, what we have demonstrated is that imbalanced diets during pregnancy generate higher rates of apoptosis that could have affected placental development and function. Our study sets the bases on placental apoptosis in rabbit placenta at term and is in line with the range of degree of apoptosis reported in previous studies of MFR [49] and placental localization in mouse [81], showing higher rates of apoptosis in the decidua with respect to the other zones. Thus, this study has corroborated that apoptosis in placenta tissue is a biological phenomenon and could be a mechanism of placental remodeling [82], that may be associated to physiological degenerative mechanisms of the rabbit placenta near term [79, 83]. However, MFR significantly increases their rates, which may have resulted in placental insufficiency and therefore in IUGR.

As expected, UNDERFED fetuses evidenced the clearest signs of IUGR, with reductions in fetal size (weight and length) and organs (brain and liver). This reduction in fetal weight induced by MFR has been previously demonstrated in other animals such as rodents [53, 84] and sheep [85]. Our data have revealed that MFR applied only during the preimplantational period did not reduce fetal weight despite that both fetal size and organogenesis were affected. The results obtained by the ratios assessments, suggest certain level of asymmetric IUGR in both MFR groups; reinforcing the innate mechanisms of vasodilation and blood shunting of the fetus to safeguard the growth of key organs like the brain (process known as “brain-sparing effect”), even at the expense of the growth of other tissues (*e*.*g*. the liver, thymus or skeletal muscle) [31, 86, 87]. The possible postnatal consequences of these prenatal adaptations induced by MFR remain unclear in our model. In previous work, rabbit offspring developed in a MFR environment evidenced altered eating, drinking and locomotor behaviors [88], which may suggest changes in brain network organization. In humans, IUGR infants with brain sparing, showed worse neurodevelopmental and behavioural outcomes than those without signs of such mechanism [87]. In rodents, as recently reviewed by Sferruzzi-Perri and Camm [12], MFR can induce early modifications in cerebral structure, contributing to late-onset diseases in the offspring. Moreover, the cost of sparing the brain reduced liver mass in both undernourished groups, which is highly important for neonatal life, as it enables fat deposition and acts as a source of growth factors and glycogen. In addition, this prenatal reduction of liver mass along with the possible hepatic gene dysregulation already reported in IUGR rats [89] could predispose the offspring to suffering from metabolic diseases in adulthood, such as obesity, insulin resistance and type-2 diabetes [90–92]. These adaptations are likely to have implications for subsequent postnatal growth and the quality of the meat as recently reviewed by Chavatte-Palmer [2, 8]. In sheep, 50% MFR resulted in offspring with increased fat deposition and altered glucose metabolism [93]. However, the most recent studies performed in rabbits, have not found differences in meat quality parameters restricting dams by 50% [94] or 75% [95].

In conclusion, the present study has helped increase awareness of the effects of MFR in gestation. The results of the present study suggest that MFR may induce strategic decisions in the mothers to safeguard their bodyweight and metabolic status at the expense of reductions in the growth of their litters. These maternal decisions are associated with moderate changes in lipid metabolism in circumscribed periods of the pregnancy (mainly during embryo development and early placental formation), but not in leptin secretion or glucidic metabolites. Thus, MFR impairs placenta development and enhances apoptotic processes, which ultimately could reduce its functionality and lastly induce IUGR. Fetuses will be reduced in size, and organogenesis can be impaired, even if the exposure only occurs during the preimplantation period. Therefore, these results should be taken into consideration when these kind of nutritional strategies were applied in livestock animals, as no clear evidence are found in the mothers, but the progeny can be affected. Furthermore, this study reinforces the use of rabbits as valid biomedical models in the perinatal field. Additional studies are needed to determine the possible consequences induced by the MFR in the offspring fitness.
